# Predictors of voluntary medical male circumcision prevalence among men aged 25-39 years in Nyanza region, Kenya: Results from the baseline survey of the TASCO study

**DOI:** 10.1371/journal.pone.0185872

**Published:** 2017-10-05

**Authors:** Elijah Odoyo-June, Kawango Agot, Jonathan M. Grund, Frankline Onchiri, Paul Musingila, Edward Mboya, Donath Emusu, Jacob Onyango, Spala Ohaga, Leonard Soo, Boaz Otieno-Nyunya

**Affiliations:** 1 Division of Global HIV & TB (DGHT), U.S. Centers for Disease Control and Prevention (CDC), Nairobi, Kenya; 2 Impact Research and Development Organization, Kisumu, Kenya; 3 Division of Global HIV & TB (DGHT), U.S. Centers for Disease Control and Prevention (CDC), Atlanta, GA, United States of America; Cardiff University, UNITED KINGDOM

## Abstract

**Introduction:**

Uptake of voluntary medical male circumcision (VMMC) as an intervention for prevention of HIV acquisition has been low among men aged ≥25 years in Nyanza region, western Kenya. We conducted a baseline survey of the prevalence and predictors of VMMC among men ages 25–39 years as part of the preparations for a cluster randomized controlled trial (cRCT) called the **Ta**rget, **S**peed and **Co**verage (TASCO) Study. The TASCO Study aimed to assess the impact of two demand creation interventions—interpersonal communication (IPC) and dedicated service outlets (DSO), delivered separately and together (IPC + DSO)—on VMMC uptake.

**Methods:**

As part of the preparatory work for implementation of the cRCT to evaluate tailored interventions to improve uptake of VMMC, we conducted a survey of men aged 25–39 years from a traditionally non-circumcising Kenyan ethnic community within non-contiguous locations selected as study sites. We determined their circumcision status, estimated the baseline circumcision prevalence and assessed predictors of being circumcised using univariate and multivariate logistic regression.

**Results:**

A total of 5,639 men were enrolled of which 2,851 (50.6%) reported being circumcised. The odds of being circumcised were greater for men with secondary education (adjusted Odds Ratio (aOR) = 1.65; 95% CI: 1.45–1.86, p<0.001), post-secondary education (aOR = 1.72; 95% CI: 1.44–2.06, p <0.001), and those employed (aOR = 1.32; 95% CI: 1.18–1.47, p <0.001). However, the odds were lower for men with a history of being married (currently married, divorced, separated, or widowed).

**Conclusion:**

Among adult men in the rural Nyanza region of Kenya, men with post-primary education and employed were more likely to be circumcised. VMMC programs should focus on specific sub-groups of men, including those aged 25–39 years who are married, divorced/separated/ widowed, and of low socio-economic status (low education and unemployed).

## Introduction

Following the results of three randomized controlled trials showing that voluntary medical male circumcision (VMMC) reduces female to male transmission of HIV by about 60% [1–3], the World Health Organization (WHO) and Joint United Nations Programme on HIV/AIDS (UNAIDS) recommended VMMC as a new HIV prevention intervention in fourteen priority countries in eastern and southern Africa, including Kenya. These countries all have high HIV prevalence and low circumcision coverage [4]. The Kenyan Ministry of Health began providing VMMC in late 2008, with a goal of increasing the proportion of circumcised men from 84% to 94% in 5 years by performing 860,000 VMMCs among males aged 15–49 years by 2013 [5]. Mathematical modeling estimated that as few as 8 VMMCs would avert 1 HIV infection in Nyanza region (former Nyanza Province) by 2025 [6]. The Kenyan national VMMC strategy concentrated on regions with low coverage of male circumcision including Nyanza region, which is the home of the traditionally non-circumcising Luo ethnic community [7].

By the end of 2015, over 1.13 million males had been circumcised in Kenya through support from the President’s Emergency Plan for AIDS Relief (PEPFAR) [8], a vast majority of these (74%) are in Nyanza region [9]. Despite the massive scale-up of VMMC in non-circumcising communities in Kenya, uptake of VMMC has been low (63%) among men aged ≥25 years as compared to men aged 15–24 years (72%) [10]. Although there has been a significant decline in the Kenyan national HIV prevalence from 7.1% in 2007 to 5.6% in 2012 among individuals aged 15–64 years, there have not been corresponding declines in Nyanza region whose HIV prevalence stands at 15.1% [11]. In addition, HIV prevalence among uncircumcised males aged 15–64 years was more than five times as high as that of circumcised males of the same age (16.9% vs. 3.1%, respectively) [12], which emphasizes the urgent need for VMMC as an HIV prevention intervention.

As recommended by WHO and UNAIDS [4], VMMC is always offered as part of a “minimum package” of complementary services including: counseling on risks, benefits, and partial protection of VMMC; provision of HIV testing services; screening and treatment of sexually transmitted infection; age-appropriate condom promotion and provision; and surgical excision of foreskin under local anesthesia [4].

Between May 2014 and September 2015, as part of the preparations for cluster randomized controlled trial (cRCT) called **Ta**rget, **S**peed and **Co**verage (TASCO) Study of VMMC scale-up, we carried out a baseline survey to assess the prevalence and predictors of being circumcised among men aged 25–39 years who were the population of focus for the TASCO VMMC demand creation trial. The study was conducted in Nyanza region of western Kenya that is predominantly occupied by a traditionally non-circumcising Luo ethnic community, whose previous data showed a low uptake of circumcision services [7, 11]. The latest Kenya AIDS Indicator Survey (KAIS 2012) showed that the uptake of VMMC services in this region was 66.0% [11]. The prevalence of reported circumcision among men aged 25–39 years in this study was 51.0%, which is lower than both the country’s and region’s estimated prevalence [10]. We therefore undertook a survey to assess factors associated with VMMC prevalence among men ages 25–39 to inform implementation of interventions to improve VMMC uptake in this specific population. This paper reports findings of the baseline survey of the TASCO Study.

## Methods

### Study design

This cross-sectional survey was conducted as part of baseline preparatory activities for a parallel arm cRCT in 11 administrative sub-counties (formerly districts) of the former Nyanza Province (now Nyanza Region), western Kenya. At baseline, prior to the implementation of the cRCT, we administered a questionnaire in local language (Dholuo) or English (see supplementary materials) in which we asked consented participants if they are circumcised; in addition, we confirmed the circumcision status of participants who consented to physical verification.

### Study sites

**Fig 1:**
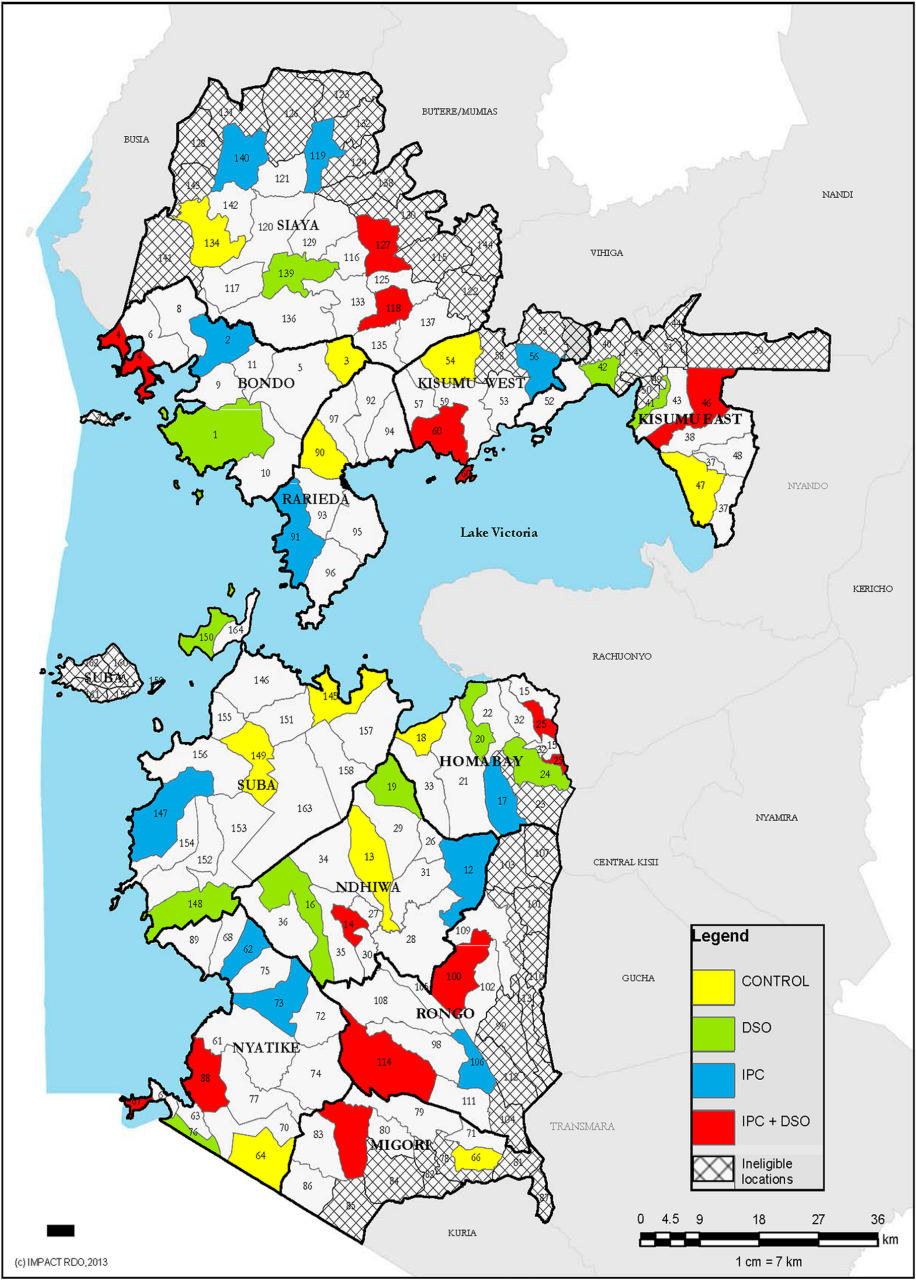
Selected study locations (clusters).

Administrative Locations (clusters) were used as the unit of randomization. Locations are the third smallest administrative units in Kenya after what were formerly known as Province, District and Division. The 11 districts comprised 164 Locations (Fig 1), 122 of which met the eligibility criteria of not participating in other VMMC demand creation or implementation science studies, not contiguous to traditionally circumcising communities, accessible by road, or not located in urban Kisumu City, as much of the city is multi-ethnic and therefore inhabited by both traditionally circumcising and non-circumcising communities. A multi-stage selection process was used to identify participating Locations (Fig 1). Participating Locations were selected one at a time from the 122 eligible ones, and contiguous Locations excluded to prevent contamination among study arms. A total of 45 non-contiguous Locations were selected and randomly assigned to one of four study arms.

### Study population

The study population included eligible men aged 25–39 years in the selected households. We conducted a complete household listing of all men aged 25–39 years in all selected villages. After an adult household member provided verbal informed consent, men aged 25–39 years were enumerated and their age and contact information were recorded. Residents were considered to be a part of a household if they lived in the same dwelling and shared food with other household members. Study eligibility also included men who intended to remain in the village for at least 9 months (which was the estimated duration of data collection).

### Enrollment and outcome

Research assistants (RAs) returned to households where men aged 25–39 years were previously enumerated during the household listing. The RAs administered written informed consent to eligible male participants and assigned them unique identification numbers. A baseline questionnaire was then administered, containing questions about circumcision status including location and approximate date of circumcision, and the top three reasons why they chose to get circumcised. For those participants reporting being uncircumcised, they also provided the top three reasons why they chose not to get circumcised. The primary outcome for this baseline survey was reported circumcision status.

### Data analysis

Baseline categorical demographic characteristics were summarized using proportions and compared between circumcised and uncircumcised men using Chi-square or Fisher’s exact tests, while continuous variables were summarized using means and standard deviation (SD) and compared using t-tests and ANOVA. Prior to conducting analyses, we had reviewed the published peer-reviewed literature to carefully identify potential variables for inclusion in the bivariate and multivariate logistic regression analyses. To account for the clustered nature of the data, we ran the generalized estimating equations (GEE) logistic regression models. We evaluated the unadjusted association of being circumcised and each candidate cofactor of interest in bivariate logistic regression model. To identify predictors of circumcision, we simultaneously included all the select cofactors in a multivariate logistic model. We did not use bivariate analysis (BVA) or significance testing to screen candidate variables for inclusion in the multivariate analysis because this approach makes strong implicit assumptions about presence or absence of confounding [12]. Data entry and management was done in a MS Access database and all analyses were done using Stata MP statistical software version 14.1 [13]. All p-values less than 0.05 were considered to be statistically significant.

### Ethical considerations

Ethical approvals were obtained from the Kenyatta National Hospital/University of Nairobi Ethics Review Committee and the Centers for Disease Control and Prevention (CDC) Institutional Review Board (protocol number 6456). The study also underwent scientific review at the CDC Office of the Associate Director of Science in the Division of Global HIV & Tuberculosis, Atlanta, Georgia. The study is registered at clinicaltrials.gov (NCT02497989).

## Results

**Fig 2:**
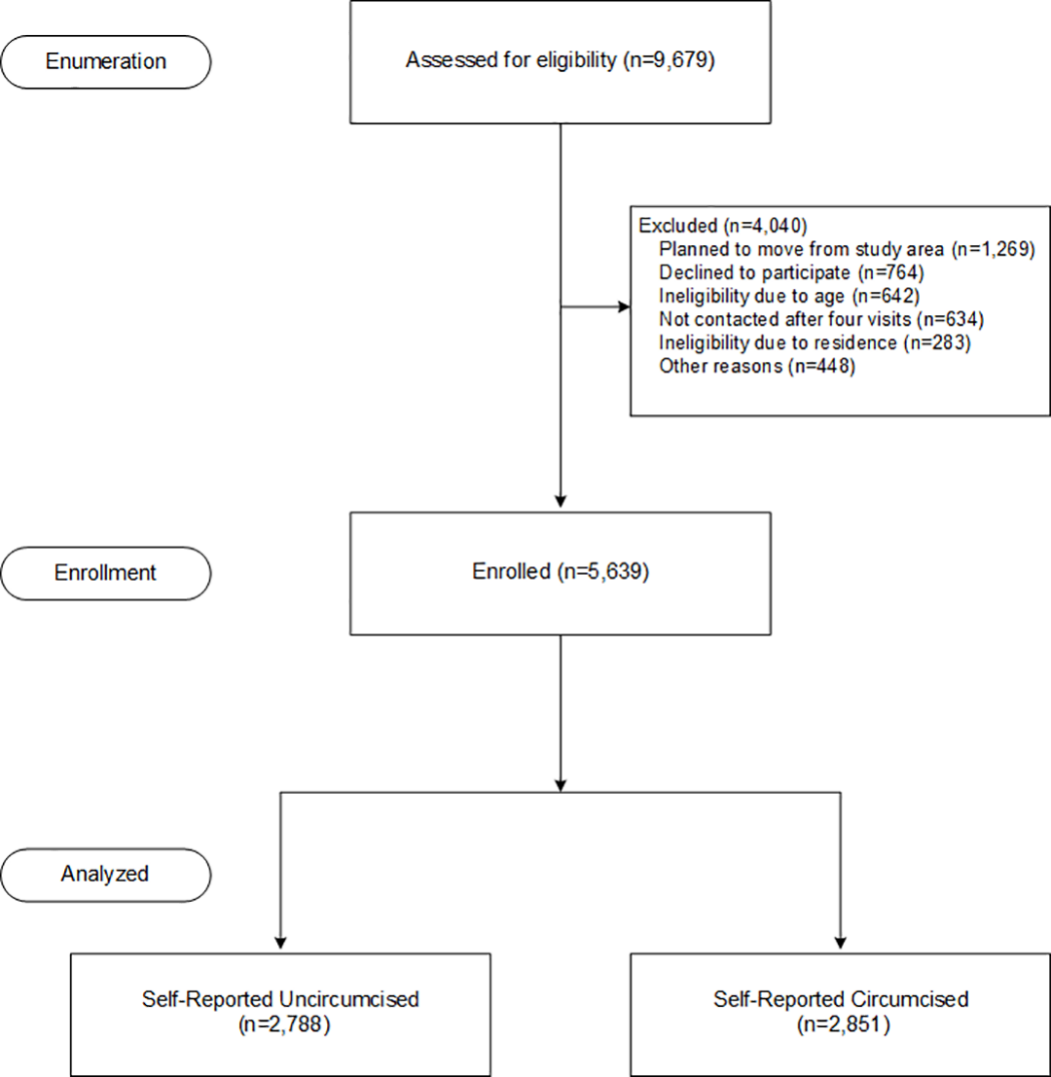
Participants enrolment flowchart.

From May 2014 to September 2015, 34,650 households were listed in 209 villages from the selected 45 locations, of which 9,238 households had 9,679 men aged 25–39 years. A total of 4,040 (41.7%) were not included in the survey for various reasons: planned to move away from the study region 31.4% (1,269), could not be contacted 15.7% (634), refused 18.9% (764), not aged 25–39 years old 15.9% (642), resided outside of study area 7.0% (283), or otherwise ineligible (deceased, illiterate, incapable of participation and independent witness unavailable) 11.1% (448). We enrolled 5,639 (58.3%) age-eligible men (Fig 2) of which 2,851 (50.6%) self-reported being circumcised. In Fig 2, we present a flowchart of participant exclusions and enrollment.

The characteristics of enrolled men are presented in Table 1. Overall, these men were on average 31.3 (SD: 4.1) years old, nearly all of them were Christians (98.7%), and a majority were married (86.2%). More than half (60.2%) had at most primary education and 62.5% were unemployed.

**Table 1:** Demographic characteristics of men 25–39 years old; overall and by circumcision status.

		Reported Circumcision status		
Factor	All (n = 5,639)	Uncircumcised (n = 2,788)	Circumcised (n = 2,851)	p-value
Age (mean (SD))	31.3 (4.1)	31.4 (4.2)	31.1 (4.0)	0.006
	n (%)	n (%)	n (%)	
**Age group**				0.001
25–29	2,234 (39.6)	1,077 (38.6)	1,157 (40.6)	
30–34	1,947 (34.5)	928 (33.3)	1,019 (35.7)	
35–39	1,458 (25.9)	783 (28.1)	675 (23.7)	
**Marital status**				<0.001
Single	646 (11.5)	278 (10.0)	368 (12.9)	
Married	4,858 (86.2)	2,427 (87.1)	2,431 (85.3)	
Divorced/separated/widowed	135 (2.4)	83 (3.0)	52 (1.8)	
**Religion**				0.008
Christian	5574 (98.8)	2,767 (99.2)	2,807 (98.5)	
Non-Christian	65 (1.2)	21 (0.8)	44 (1.5)	
**Education completed**				<0.001
Primary	3,397 (60.2)	1,868 (67.0)	1529 (53.6)	
Secondary	1,613 (28.6)	671 (24.1)	942 (33.0)	
Post-Secondary	629 (11.2)	249 (8.9)	380 (13.3)	
**Employment status**				<0.001
Unemployed	2,114 (37.5)	1,154 (41.4)	960 (33.7)	
Employed	3,525 (62.5)	1,634 (58.6)	1891 (66.3)	

Men who were circumcised were significantly different from uncircumcised with respect to age, marital status, religion, education and employment status (Table 1). However, those circumcised were more likely than the uncircumcised to have more than primary level education and to be employed.

Independent factors significantly associated with being circumcised included non-Christian religion, adjusted odds ratio (aOR = 2.01, 95% CI 1.20–3.47), secondary education (aOR = 1.65, 95% CI 1.45–1.86) or post-secondary education (aOR = 1.72, 95% CI 1.44–2.06) and being employed (aOR = 1.32, 95% CI 1.18–1.47). The odds of being circumcised were significantly lower for men aged 35–39 (aOR = 0.85, 95% CI 0.74–0.98) and those married (OR = 0.84; 95% CI: 0.70–1.00) and 41% lower for divorced/separated/widowed (OR = 0.59; 95% CI: 0.39–0.87) men (Table 2).

**Table 2:** Predictors of being circumcised among men aged 25–39 years.

		Circumcision Prevalence	Univariate Analysis			Multivariate Analysis		
	Covariate		^†^OR	95% ^#^CI	p-value	^‡^aOR	95% CI	p-value
								
**Age group**								
	25–29*	51.8%	1			1		
	30–34	52.3%	1.02	(0.91–1.15)	0.724	1.08	(0.95–1.23)	0.225
	35–39	46.3%	0.80	(0.70–0.92)	0.001	0.85	(0.74–0.98)	0.023
**Marital Status**								
	Single*	57.0%	1			1		
	Married	50.0%	0.76	(0.64–0.89)	<0.001	0.84	(0.70–1.00)	0.050
	Divorced/Separated/Widowed	38.5%	0.47	(0.32–0.69)	<0.001	0.59	(0.39–0.87)	0.008
**Religion**								
	Christian*	50.4%	1			1		
	Non-Christians	67.7%	2.07	(1.24–3.55)	0.007	2.01	(1.20–3.47)	0.010
**Education**								
	Primary*	45.0%	1			1		
	Secondary	58.4%	1.72	(1.52–1.93)	<0.001	1.65	(1.45–1.86)	<0.001
	Post-Secondary	60.4%	1.86	(1.57–2.22)	<0.001	1.72	(1.44–2.06)	<0.001
**Employment Status**								
	Unemployed*	53.6%	1			1		
	Employed	45.4%	1.39	(1.25–1.55)	<0.001	1.32	(1.18–1.47)	<0.001

† = Odds ratio (OR)

‡ = Adjusted odds ratio; (aOR)

# = Confidence interval

* = Referent group.

## Discussion

Our survey of the prevalence and predictors of reported circumcision among men ages 25–39 years in a Kenyan region occupied mainly by a traditionally non-circumcising ethnic community, found that only half (50.6%) of these men were circumcised. Predictors of being circumcised included being non-Christian, post-primary education, and being employed. Post-primary education level as a predictor of circumcision is in line with previous studies that reported that better knowledge about the benefits of male circumcision was positively associated with its acceptability [14, 15]. Similarly, a study in South Africa demonstrated association between higher education and male circumcision acceptability [16].

The finding that men who were employed have greater odds of being circumcised compared to their unemployed counterparts is corroborated by a study in Kenya, which showed that men who were in formal employment earning salaries did not express loss of income as a barrier to uptake of VMMC, whereas those earning a daily wage expressed concern about financial loss [17]. Being employed as a predictor for being currently circumcised could therefore be attributed to a more stable income that could afford an individual time off to heal without worrying about loss of income.

In this study, being married was significantly associated with being uncircumcised. This corroborates the finding of a study in Zimbabwe that reported that partner refusal was a major VMMC barrier due to the spousal concern about the justification for VMMC for HIV prevention among a married couple [18]. These women found it difficult to envisage how HIV transmission would take place within a married couple, unless the man intended to become unfaithful. Moreover, there was concern about the long abstinence period of six weeks post-circumcision [18]. This finding suggests the influence an intimate partner can have on male partner's decision to accept or decline circumcision.

On the other hand, a household survey done in Zambia in 2007, showed that 90.8% of the women participants were in favor of their male partner undergoing VMMC as an effective method of HIV prevention [19]. In addition to reducing HIV, VMMC has been shown to reduce the risk of Human Papilloma Virus associated with cervical cancer in female partners of circumcised men [20]. This suggests that putting in place VMMC demand creation strategies that involves women will increase their awareness and could promote support for their partners and sons to get circumcised [21]. Future demand creation efforts for VMMC should therefore focus on females with accurate information on the benefits of VMMC to both men and women and involve them in encouraging their spouses to go for VMMC. Another special group for consideration in future demand creation interventions are formerly married men (i.e., those who self-reported being divorced, separated, or widowed). Formerly married men had a lower prevalence of circumcision than single or married men, even after adjusting for age. This is a subgroup that may be willing to become circumcised and is likely to be at higher risk of HIV than currently married men.

The study had some limitations including the fact that some men who were enumerated were not enrolled in the study mainly due to out-migration from the study area. Additionally, the study findings have generalizability limited only to non-circumcising community in Nyanza region.

This study identified the predictors of circumcision prevalence among men aged 25–39 years in Nyanza region, Kenya, which are essential for informing strategies to increase uptake of voluntary medical male circumcision in the region. Specifically, demand creation strategies are needed that focus on and appeal to older men who have low level of education, are formerly married, and are unemployed.

## Supporting information

S1 Questionnaire Enrollment questionnaire and circumcision verification at baseline and endline-English. (DOC)

S2 Questionnaire Enrollment questionnaire and circumcision verification at baseline and endline-Dholuo. (DOC)

S1 Dataset VMMC predictor dataset. (XLSX)
