# Supplementary material for: Predictors of voluntary medical male circumcision prevalence among men aged 25-39 years in Nyanza region, Kenya: Results from the baseline survey of the TASCO study
Source: PLoS One. 2017 Oct 5;12(10):e0185872. doi: 10.1371/journal.pone.0185872 (PMC5628861; doi:10.1371/journal.pone.0185872)
Supplement: S1 Questionnaire — (DOC) [file pone.0185872.s001.doc]

Participant Unique ID#: ___________________________

District: ______________ Location/Cluster: _______________ Service site: _____________________

***Instructions to Study Staff (Counselor)***

**Step 1: Obtain written informed consent** (*using Appendix 2A at baseline and Appendix 2C at endline*)

**Step 2: Conduct interview before circumcision verification**

**Step 3: Verify circumcision status if consent if provided**

**Participant Demographic Information (*at Baseline only*)**

Age: _____________ ***(in years at last birthday)***

Religion: 🞏 Christian 🞏 Islam 🞏 Other ___________________ 🞏 None

Highest education level: _____________________ ***(none, primary, secondary, college, university)***

Employment Status: 🞏 Unemployed 🞏 Wage 🞏 Salaried

Marital status: 🞏 Single 🞏 Married (monogamous) 🞏 Married (polygamous) 🞏 Widowed

🞏 Divorced/Separated

**CIRCUMCISION STATUS QUESTIONS AND VERIFICATION *(at baseline and enndline)***

*Now, I am going to ask you a few questions about your circumcision*

Are you circumcised? |____| Yes |____| No

If yes, where and when were you circumcised?

Where? ________________ (site/facility) When? __ __ / __ __ __ /__ __ __ __

**Uncircumcised men:** Can you give us three main reasons why you have not gone for VMMC? *[Do not read out; circle all responses, then ask client for top 3 reasons and write rank next to choice]*

| ***Reasons*** | ***Rank*** | ***Reasons*** | ***Rank*** | ***Reasons*** | ***Rank*** |
| --- | --- | --- | --- | --- | --- |
| Reduce sexual function |  | Penis will shrink/get deformed |  | What I/family will eat |  |
| Increase sexual function |  | Contrary to culture/religion |  | I am not at risk for HIV/STI |  |
| Female partner opposed |  | Line up with young people |  | Time/venue not convenient |  |
| Family opposed |  | Served by female providers |  | VMMC is for younger people |  |
| Peers opposed |  | Served by young providers |  | Waiting time too long |  |
| Pain |  | Sexual abstinence period long |  | Other – specify __________ |  |

**Circumcised men:** Can you give us three main reasons why you went for VMMC? *[Do not read out; circle all responses, then ask client for top 3 reasons and write rank next to choice]*

| ***Reasons*** | ***Rank*** | ***Reasons*** | ***Rank*** | | ***Reasons*** | ***Rank*** |
| --- | --- | --- | --- | --- | --- | --- |
| Reduce HIV risk |  | Partner encouraged |  | Improve sexual performance | |  |
| Improve genital hygiene |  | Parents encouraged |  | Looks prettier | |  |
| Reduce STI risk |  | Friends encouraged |  | Short waiting time | |  |
| Reduce penile cancer |  | Workmates encouraged |  | Other – specify _____________ | |  |
| Reduce cervical cancer in partner |  | Teachers encouraged |  | Other – specify _____________ | |  |
| Easy to use condom |  | Fit in MC communities |  | Other – specify _____________ | |  |
| Culture/religion |  | Influenced by mobilizer |  | Other – specify _____________ | |  |

***If participant gave consent (Appendix 2A at baseline) for visual inspection of the penis to categorize the circumcision status, proceed to inspect the penis at the preferred location available. Ensure there is privacy. At endline verify circumcision status only of men who report having been circumcised and who consent to visual inspection using Appendix 2C.***

| **Findings on Visual Inspection (in a flaccid state)** |
| --- |
| |____| Fully circumcised: No foreskin.  |____| Partially circumcised: Foreskin is past corona sulcus but covers less than one half of the glans.  |____| Uncircumcised: Foreskin covers one half or more of the glans. |

(Thank the client and assure him of confidentiality; answer questions if any)
