# Supplementary material for: Predictors of voluntary medical male circumcision prevalence among men aged 25-39 years in Nyanza region, Kenya: Results from the baseline survey of the TASCO study
Source: PLoS One. 2017 Oct 5;12(10):e0185872. doi: 10.1371/journal.pone.0185872 (PMC5628861; doi:10.1371/journal.pone.0185872)
Supplement: S2 Questionnaire — (DOC) [file pone.0185872.s002.doc]

Participant Unique ID#: ___________________________

District: ______________ Location/Cluster: _______________ Service site: _____________________

***Instructions to Study Staff (Counselor)***

**Step 1: Obtain written informed consent** (*using Appendix 2A at baseline and Appendix 2C at endline*)

**Step 2: Conduct interview before circumcision verification**

**Step 3: Verify circumcision status if consent if provided**

**Participant Demographic Information (*at Baseline only*)**

Higni: _____________ ***(e higni e nyasi mar paro nyuolne mogik)***

Dini: 🞏 Jakristo 🞏 Ja Islamm 🞏 Mamoko ___________________ 🞏 Onge

Highest education level: _____________________ ***(none, primary, secondary, college, university)***

Chal mar tich: 🞏 Ok ondike 🞏 Ichule suku 🞏 Ichule osara

Chal mar keny: 🞏 Ok okendo 🞏 Okendo jaot achiel) 🞏 Jadoho 🞏 Jaodo osetho

🞏 Owere/SeparatedOpogore gi jaot

**CIRCUMCISION STATUS QUESTIONS AND VERIFICATION *(at baseline and enndline)***

*Koro, adwaro penji penjo matin ewi nyangu mari.*

Be osetimni nyangu? |____| Ee |____| Ooyo

Ka Ee, kanye to karng’o ma ne otimni nyangu?

Kanye? ________________ (site/facility) Karang’o? __ __ / __ __ __ /__ __ __ __

**Chuo ma ok otimnegi nyangu:** Bende inyalo nyisa gigo adek moloyo ma omiyo pok idhi mondo otimni nyangu chuo ma itimo e yo thieth e hero ng’ato kata VMMC? *[Do not read out; circle all responses, then ask client for top 3 reasons and write rank next to choice]*

| ***Reasons*** | ***Rank*** | ***Reasons*** | ***Rank*** | ***Reasons*** | ***Rank*** |
| --- | --- | --- | --- | --- | --- |
| Duok chien teko mar bedo e achiel e ringruok |  | Duong’na biro ng’wony/nenore march |  | Gima an kata joot biro chamo |  |
| Medo teko mar bedo e achiel e ringruok |  | Ok en chikwa/ok en chik dinina |  | Aonge e thuolo mar gamo kute mag Ayaki kata nyach |  |
| Jaherana ma miyo ne ok dwar |  | Bedo e laini gi rowere |  | Saa kata kama itime nynagu ok ber koda |  |
| Joot ok dwar |  | Jochiw kony ma mine |  | Nyangu en mar joma rowere |  |
| Osiepe ok dwar |  | Jochiw kony ma rowere |  | Saa ma irito bor ahinya |  |
| Rem |  | Kinde mar thegruok gi bedo e achiel e ringruok bor |  | Ma moko – ler __________ |  |

**Chuo ma otimnegi nyangu:** Bende inyalo nyisa gigo adek moloyo ma omiyo ne idhi mondo otimni nyangu chuo ma itimo e yo thieth e hero ng’ato kata VMMC? *[Do not read out; circle all responses, then ask client for top 3 reasons and write rank next to choice]*

| ***Reasons*** | ***Rank*** | ***Reasons*** | ***Rank*** | | ***Reasons*** | ***Rank*** |
| --- | --- | --- | --- | --- | --- | --- |
| Duoko chien thuolo mar gamo kute mag Ayuaki |  | Jahera ne ojiwa |  | Medo ber bedo e achiel e achiel ringruok | |  |
| Omedo ler mar duong’ dichuio |  | Jonyuol ne ojiwa |  | Nenore majaber | |  |
| Duoko chien thuolo mar gamo nyaye |  | Osiepe ne ojiwa |  | Saa mar rito tin | |  |
| Duoko chien nwang’o kansa mar duong’ dichuo |  | Jotichwa ne ojiwa |  | Ma moko – ler __________ | |  |
| Duoko chien nwang’o kansa mardho ofuku nyuol mar jahera |  | Japuonj ne ojiwa |  | Ma moko – ler __________ | |  |
| Yot tiyo gi raboyunga |  | Mondo abed achiel kuom joma osetimnegi nyangu |  | Ma moko – ler __________ | |  |
| Kitwa/dini |  | Ja luom ji ne olondha |  | Ma moko – ler __________ | |  |

***If participant gave consent (Appendix 2A at baseline) for visual inspection of the penis to categorize the circumcision status, proceed to inspect the penis at the preferred location available. Ensure there is privacy. At endline verify circumcision status only of men who report having been circumcised and who consent to visual inspection using Appendix 2C.***

| **Findings on Visual Inspection (in a flaccid state)** |
| --- |
| |____| Fully circumcised: No foreskin.  |____| Partially circumcised: Foreskin is past corona sulcus but covers less than one half of the glans.  |____| Uncircumcised: Foreskin covers one half or more of the glans. |

(Thank the client and assure him of confidentiality; answer questions if any)
